# The Role of Emotion Regulation and Loss-Related Coping Self-efficacy in an Internet Intervention for Grief: Mediation Analysis

**DOI:** 10.2196/27707

**Published:** 2022-05-06

**Authors:** Jeannette Brodbeck, Thomas Berger, Nicola Biesold, Franziska Rockstroh, Stefanie J Schmidt, Hansjoerg Znoj

**Affiliations:** 1 Department of Clinical Psychology and Psychotherapy University of Bern Bern Switzerland; 2 School of Social Work University of Applied Sciences and Arts Northwestern Switzerland Olten Switzerland; 3 University Hospital of Child and Adolescent Psychiatry and Psychotherapy Bern Switzerland; 4 Department of Health Psychology University of Bern Bern Switzerland

**Keywords:** grief, divorce, separation, bereavement, internet intervention, emotion regulation, coping self-efficacy, mediation

## Abstract

**Background:**

Internet interventions for mental disorders and psychological problems such as prolonged grief have established their efficacy. However, little is known about how internet interventions work and the mechanisms through which they are linked to the outcomes.

**Objective:**

As a first step in identifying mechanisms of change, this study aimed to examine emotion regulation and loss-related coping self-efficacy as putative mediators in a randomized controlled trial of a guided internet intervention for prolonged grief symptoms after spousal bereavement or separation or divorce.

**Methods:**

The sample comprised older adults who reported prolonged grief or adaptation problems after bereavement, separation, or divorce and sought help from a guided internet intervention. They were recruited mainly via newspaper articles. The outcome variables were grief symptoms assessed using the Texas Revised Inventory of Grief and psychopathology symptoms assessed using the Brief Symptom Inventory. A total of 6 module-related items assessed loss-focused emotion regulation and loss-related coping self-efficacy. In the first step, path models were used to examine emotion regulation and loss-related coping self-efficacy as single mediators for improvements in grief and psychopathology symptoms. Subsequently, exploratory path models with the simultaneous inclusion of emotion regulation and self-efficacy were used to investigate the specificity and relative strength of these variables as parallel mediators.

**Results:**

A total of 100 participants took part in the guided internet intervention. The average age was 51.11 (SD 13.60) years; 80% (80/100) were separated or divorced, 69% (69/100) were female, and 76% (76/100) were of Swiss origin. The internet intervention increased emotion regulation skills (β=.33; *P*=.001) and loss-related coping self-efficacy (β=.30; *P*=.002), both of which correlated with improvements in grief and psychopathology symptoms. Path models suggested that emotion regulation and loss-related coping self-efficacy were mediators for improvement in grief. Emotion regulation showed a significant indirect effect (β=.13; *P*=.009), whereas coping self-efficacy showed a trend (β=.07; *P*=.06). Both were confirmed as mediators for psychopathology (β=.12, *P*=.02; β=.10; *P*=.02, respectively). The path from the intervention to the improvement in grief remained significant when including the mediators (β=.26, *P*=.004; β=.32, *P*≤.001, respectively) in contrast to the path from the intervention to improvements in psychopathology (β=.15, *P*=.13; β=.16, *P*=.10, respectively).

**Conclusions:**

Emotion regulation and loss-related coping self-efficacy are promising therapeutic targets for optimizing internet interventions for grief. Both should be further examined as transdiagnostic or disorder-specific putative mediators in internet interventions for other disorders.

**Trial Registration:**

ClinicalTrials.gov NCT02900534; https://clinicaltrials.gov/ct2/show/NCT02900534

**International Registered Report Identifier (IRRID):**

RR2-10.1186/s13063-016-1759-5

## Introduction

### Background

Several studies and meta-analyses have established the efficacy of traditional face-to-face interventions for grief counseling [1,2]. In addition, guided internet interventions for prolonged grief symptoms have demonstrated their efficacy with effect sizes in the moderate to large range [3-6]. A recent review and meta-analysis concluded that internet interventions for bereaved individuals with higher levels of disturbed grief showed medium effect sizes, suggesting that further research should focus on the moderators and underlying mechanisms of treatments [7]. The components of grief counseling mostly provided in palliative care settings were psychoeducation, enhancing communication and social support, stress reduction/relaxation skills, cognitive reframing, and identifying and modifying maladaptive behaviors [8]. Psychological interventions for severe, prolonged grief focus on cognitive restructuring, exposure, interpersonal elements, and behavioral activation [9]. A component of most internet interventions for grief is expressive writing assignments, which can foster the emotional processing of the loss, may lead to new perspectives on the loss, and might foster the process of sense making [7].

Although considerable evidence has established the efficacy of cognitive behavioral (internet) interventions for psychiatric disorders and several emotional problems, including grief, little is known about *how* they lead to an improvement in symptoms or behavior [10,11]. The identification of these mechanisms of change would be useful for tailoring interventions that specifically target these mechanisms and thus may be more potent or efficient [12]. It may also contribute to the development of more parsimonious interventions with fewer but equally effective components [13], which reduce the burden for clients as well as save time and cost [14].

Potential mechanisms of change can be specific factors posited in the theoretical background of the intervention (eg, changes in maladaptive thinking or behavior in cognitive behavioral therapy [CBT]) or common factors, such as the therapeutic alliance, empathy, expectations, or a rationale that provides credibility to the intervention [15]. Self-efficacy is a central variable in social cognitive theories [16]. Self-efficacy as a belief in the ability to exercise control over events that affect one’s life to manage one’s personal functioning and environmental demands plays an important role in stress reactions and adaptive coping in threatening situations [17]. Bereavement coping self-efficacy (CSE) predicted lower emotional distress, higher psychological and spiritual well-being, and better physical health in widows whose husbands had died of cancer [18]. Self-efficacy also predicted lower grief symptoms in students who lost a close person in a university campus shooting [19]. In addition, Benight and Bandura [17] concluded that CSE was a mediator in the recovery from traumatic experiences. In line with this notion, a change in CSE predicted a decrease in posttraumatic stress symptoms in an eHealth intervention for survivors of trauma [20]. Moreover, self-efficacy was a mediator between psychopathology symptoms and disabilities in activities and participation [21].

Emotion regulation (ER) has been established as a transdiagnostic risk factor for different psychological disorders [22] and is a central intervention target in psychotherapy [23]. A review concluded that face-to-face ER interventions had positive short- and long-term effects on emotion process outcomes, affect and mood states, and medical and psychiatric disorders [24]. Improvements in the ability to modify, accept, and tolerate negative emotions were consistent predictors of treatment outcomes in patients with various mental disorders [23]. Moreover, CBT enriched with ER training resulted in a greater reduction in depression and negative affect and increased well-being than routine CBT [25].

Although some studies have examined ER training as a predictor or outcome of treatment, few studies have investigated ER as a mechanism of change. For example, the modification of negative emotions was found to mediate the link between ER skills and psychopathological symptoms assessed using the Brief Symptom Inventory [26]. Furthermore, ER was a mediator and putative mechanism of change in an internet intervention for stress management [27].

ER and loss-related CSE can be integrated as putative mechanisms of change in the existing models of coping with grief. The dual process model of coping with bereavement posits that loss-oriented tasks, such as grief work, experiencing the pain of the loss, expressing emotions toward the deceased, and transforming bonds with the lost person, are necessary for positive adaptation to the loss [28]. Similarly, the task model of mourning specifies tasks such as accepting the reality of the loss and experiencing the pain of grief [29]. Improvement in ER may be especially important for these loss-related tasks [30,31]. ER skills may make these processes more tolerable by facilitating the modulation of overwhelming or more persistent painful emotions.

Furthermore, the dual process model describes the importance of restoration-oriented tasks such as engaging in new activities and finding new social roles and identities. Restoration-oriented tasks can be perceived as very stressful, and a high level of loss-related CSE and the belief in the ability to achieve these goals may facilitate tackling these tasks and increase the sense of autonomy, self-determination, purpose in life, and perceived environmental mastery, leading to less avoidant behavior and less aversive rumination [18]. High loss-related CSE may decrease the appraisal of these restoration-oriented tasks as threatening, alleviate stress and anxiety, promote engagement in coping behavior, and sustain coping efforts [17]. Thus, loss-related CSE could promote positive adaptation to a life without the partner and decrease loss-related symptoms.

As a third element, the dual process model highlights the importance of oscillating between loss- and restoration-oriented tasks. ER skills may not only foster coping with loss-oriented tasks but also the alternation between loss and restoration orientation by eventually limiting grief work, rumination, and pain and facilitating distraction, soothing, and cheering oneself up.

### Objectives

This study examines ER and loss-related CSE as potential mechanisms of change in an internet intervention, called LIVIA, for prolonged grief symptoms after spousal bereavement, separation, or divorce [32,33]. LIVIA addressed older adults who had experienced spousal bereavement, separation, or divorce and sought help for coping with prolonged grief symptoms, psychological distress, or adaptation problems in daily life. Thus, LIVIA is the first intervention that focuses on grief after bereavement, as well as grief after separation or divorce. Both events require similar adaptation and mourning tasks identified by Worden [29]; that is, accepting the reality of the loss, processing the pain of grief, adjusting to a life without the spouse, and remembering the lost spouse while reinvesting emotional energy into a new life. We assume that the dual process model is also applicable for separation or divorce from a spouse insofar as these events, similar to bereavement, imply breaking the bond and necessarily lead to the reorganization of one’s life circumstances. Furthermore, we hypothesize that the effect of the intervention is based on the same mechanisms of change.

The comparison of baseline characteristics and the efficacy of LIVIA for widowed and divorced participants, as well as the stability of the effects over 3 months, have been described elsewhere [33]. LIVIA improved grief, depression symptoms, psychopathological distress, embitterment, loneliness, and life satisfaction compared with the waitlist group. The between-group effect sizes were *d*=0.81 for grief and *d*=0.39 for psychopathology symptoms.

Building on these results, the present post hoc analyses aim to investigate whether gains in ER skills and loss-related CSE mediated the intervention effects as a first step in elucidating mechanisms of change in an intervention for grief after spousal bereavement, separation, or divorce. This study is one of the few to investigate mediators in internet interventions and the first to examine emotional and cognitive processes as mediators in a grief intervention. We hypothesize that both gains in ER skills and loss-related CSE mediated the effect of the intervention on improvements in grief and psychopathology symptoms.

## Methods

### Recruitment

The data presented in this study were based on a randomized controlled trial that evaluated the efficacy of LIVIA compared with a waitlist control group (ClinicalTrials.gov NCT02900534). Participants were mainly recruited via newspaper articles and web-based self-help forums. The main inclusion criteria were the experience of spousal bereavement, separation, or divorce >6 months before enrolling in the study and seeking help to cope with prolonged grief symptoms, psychological distress, or psychosocial adaptation to a life without a partner. The main exclusion criteria were severe psychological or somatic disorders that needed immediate treatment, acute suicidality (Beck Depression Inventory suicide item >1 or suicidal ideation in the telephone interview), concomitant psychotherapy, and/or prescribed drugs against depression or anxiety if prescription or dosage had changed in the month before or during the internet intervention.

### Participants

Of the total sample of 110 individuals, 9 (8.2%) individuals did not start the internet intervention and were excluded from the present analyses. One of the participants was excluded because of being a multivariate outlier, which affected the mediation analyses. Therefore, the analysis sample comprised 100 German-speaking participants who lost their spouse through bereavement (20/100, 20%), separation, or divorce (80/100, 80%) and who were randomly allocated to the intervention group or the waitlist control group. The waitlist control group received access to the treatment after 12 weeks. The participants first provided electronic and then oral informed consent in a telephone screening interview.

### Ethics Approval

This study was approved by the Cantonal Ethics Committee of the Canton of Bern, Switzerland (BASEC2016-00180).

### Measures

The severity of grief symptoms was assessed using the 16-item Texas Revised Inventory of Grief–German Version (TRIG) [34]. The answer categories ranged from 1=*completely true* to 5=*completely false*. Cronbach α was .86 in the preintervention measurement and .90 in the postintervention measurement. The TRIG includes items that are applicable after divorce and bereavement and has proven to have good factorial validity that was temporally invariant over 1 year [35].

Psychopathology symptoms were measured using the German version of the widely used Brief Symptom Inventory [36]. The 53 items assessed a broad range of somatic and psychopathological symptoms within 7 days before completing the questionnaire. Answer categories ranged from 0=*not at all* to 4=*very much*. Cronbach alpha was .90 in the preintervention measurement and .96 in the postintervention measurement.

A total of 6 module-related items assessed loss-focused ER and loss-related CSE. The response categories ranged from −3=*not at all* to 3=*yes, exactly*. A confirmatory factor analysis, including the 6 items, supported a 2-factor model compared with a 1-factor model (1-factor model: Comparative Fit Index=0.84, Tucker-Lewis Index=0.74, root mean square error of approximation=0.273; 2-factor model: Comparative Fit Index=0.95, Tucker-Lewis Index=0.91, root mean square error of approximation=0.159). The details on the development of these measures and the results of the exploratory and confirmatory factor analyses are presented in Multimedia Appendix 1. Loss-focused ER was assessed with the following module-related items: “I can cheer myself up,” “I can have a positive influence on my thoughts and feelings,” and “I can take care of my own well-being.” Loss-focused CSE contained 3 items: “I am convinced that I can cope with the loss of my spouse/with the separation or the divorce,” “I am ready to do what is necessary to overcome my loss,” “I have a strong influence on the coping with my loss.” Cronbach α for ER was .90 in the preintervention measurement and .91 in the postintervention measurement, and Cronbach α for self-efficacy was .76 in the preintervention measurement and .85 in the postintervention measurement. To measure gains in ER and self-efficacy, we subtracted the presum score from the postsum score. Thus, a positive value indicated a gain during the intervention. All self-report questionnaires were web-based using Qualtrics (QualtricsXM) [37] at baseline (ie, before the intervention) and after the intervention 12 weeks after receiving access to the program.

In addition to the self-report questionnaires, the initial screening process included a telephone call, in which trained email supporters assessed the criteria for the Diagnostic and Statistical Manual of Mental Disorders, Fifth Edition diagnosis of persistent complex bereavement disorder. This required an adaptation of the criteria to the purpose of our study; that is, we assessed the persistence of the symptoms 6 months after the loss instead of 12 months and also used interviews with individuals who lost their spouse through separation or divorce.

### Statistical Analyses

As the first step, we computed correlations using the pooled data of the intervention and waitlist control groups, who received access to the intervention after a 12-week waiting period. The pooled data set represented a more comprehensive sample and provided a bigger sample size. In the second step, we computed mediation models for improvements in grief and psychopathology symptoms first with a single mediator and then with 2 parallel mediators in a path-analytic framework using Mplus (version 8.4) [38] using the original data set. As mediator variables, we included gains in ER and gains in loss-related CSE from the pre- to postmeasurement time points. We used observed difference scores for changes in the mediators as well as in the outcome variables (ie, grief and psychopathology symptoms). Positive change scores indicated improvements in grief and psychopathology symptoms. We used the *model indirect* command to specify and estimate the specific indirect effects for both mediators and the total indirect effect. Regarding effect sizes for direct effects, we considered standardized regression coefficients of 0.1 as small, 0.3 as medium, and 0.5 as large [39]. For indirect effects in the mediation models, we considered 0.01 as small, 0.09 as medium, and 0.25 as large effects [40].

As data were missing at random (see the following sections), we used multiple imputation to deal with missing data [41]. Multiple imputation using the Bayes estimator yielded inconsistent estimates depending on the number of iterations. Therefore, we used a robust maximum likelihood estimator to impute missing data in 100 data sets. Sensitivity analyses showed robust findings for analyses with complete cases and imputed data.

### LIVIA Intervention

The dual process model of coping with bereavement and the task model of mourning provided the theoretical background for a guided internet-based self-help intervention called LIVIA [32,33]*.* It comprised 10 text-based modules and a weekly email as guidance. The modules contained writing tasks for exposure to loss and assignments for practice in daily life. Several modules directly targeted ER processes: 3 modules focused on cognitive behavioral techniques fostering positive emotions, self-care, and social relationships, whereas 2 modules focused on exposure and loss-oriented interventions (ie, writing tasks for accepting memories and pain as well as addressing unfinished business). Loss-related CSE was a direct target in the modules, including information about grief or separation reactions, coping strategies, and restoration-oriented interventions for creating a life without the partner.

## Results

### Sample Characteristics

The mean age of the participants was 51.11 (SD 13.60, range 20-85) years, and 69% (69/100) were female. Most participants were of Swiss origin (76/100, 76%) and went to a vocational school (37/100, 37%) or university (34/100, 34%). The average time since the bereavement, separation, or divorce was 2 (SD 3.0, range 0.5-25) years, and 25% (25/100) of the participants fulfilled the B, C, and D criteria of a persistent complex bereavement disorder in the Diagnostic and Statistical Manual of Mental Disorders, Fifth Edition. Table 1 presents the baseline characteristics of the intervention group, the waitlist control group, and the pooled data sets, as well as the results of the comparison between the intervention and the control groups.

On average, participants completed 8 of the 10 modules (mean 8.03, SD 2.79), and 57% (57/100) of participants completed all modules. Completers and individuals who did not fill out the postquestionnaires did not significantly differ in terms of baseline characteristics such as demographics or level of distress (*P*>.21). However, participants who did not fill out the postquestionnaires completed significantly fewer modules than completers (mean*_Do_* 4.19, SD*_Do_* 2.81 vs mean*_C_* 8.76, SD*_C_* 2.12; *t*_98_=7.49; *P*<.001; *d*=2.04) and were significantly younger (mean*_Do_* 43.25, SD*_Do_* 14.58 vs mean*_C_* 52.63, SD*_C_* 12.95; *t*_97_=2.60; *P*<.001; *d*=0.71). This suggests a missing at random mechanism (ie, that missingness is related to measured variables in the analysis model [42]).

**Table 1:** Demographics and sample characteristics at baseline and means of grief and psychopathology at the postmeasurement time point (N=100).

Characteristics			Pooled		Intervention (n=58)		Control (n=42)		*P* value^a^
Age (years), mean (SD)			51.11 (13.60)		50.85 (12.90)		51.48 (14.68)		.83
**Gender, n (%)**									
	Female	69 (69)		43 (74)		26 (62)		.19	
	Male	31 (31)		15 (26)		16 (38)		.19	
**Event, n (%)**									
	Spousal bereavement	20 (20)		11 (19)		9 (21)		.76	
	Separation or divorce	80 (80)		47 (81)		33 (79)		.76	
**Education, n (%)**									
	Compulsory school	2 (2.)		1 (2)		1 (2)		.35	
	Apprenticeship	19 (19)		14 (24)		5 (12)		.35	
	Secondary 2	7 (7)		3 (5)		4 (10)		.35	
	Vocational school	37 (37)		19 (33)		18 (43)		.35	
	University	34 (34)		21 (36)		13 (31)		.35	
**Nationality, n (%)**									
	Swiss	76 (76)		46 (79)		30 (71)		.66	
	German-speaking countries	18 (18)		9 (16)		9 (21)		.66	
	Other countries	6 (6)		3 (5)		3 (7)		.66	
Time since event (years), mean (SD)			2.21 (3.0)		2.16 (3.47)		2.27 (2.26)		.85
Persistent complex bereavement disorder, n (%)			25 (25)		15 (26)		10 (24)		.82
**Grief, mean (SD)**									
	Before treatment	3.39 (0.78)		3.48 (0.74)		3.26 (0.82)		.18	
	After treatment	2.84 (0.89)		2.80 (0.86)		2.90 (0.93)		.60	
**Psychopathology, mean (SD)**									
	Before treatment	0.86 (0.57)		0.95 (0.63)		0.73 (0.44)		.06	
	After treatment	0.62 (0.50)		0.61 (0.51)		0.64 (0.48)		.81	

^a^Comparison between intervention and control groups; *t* tests were 2-tailed.

### Correlations Between Gains in ER and CSE, Outcomes, and Demographics

Table 2 presents the correlation matrix of predictors and outcome variables based on the pooled data set.

Baseline levels of grief and psychopathology symptoms correlated at baseline (*r*=0.48; *P*<.001), and changes in grief symptoms correlated significantly with changes in psychopathology symptoms (*r*=0.35; *P*<.001). The pre-post correlation for grief was *r*=0.68, and the pre-post correlation for psychopathology symptoms was *r*=0.64 (both *P*<.001). Gains in ER correlated with gains in loss-related CSE (*r*=0.45; *P*<.001). Gains in ER and CSE correlated with improvements in grief symptoms and psychopathology symptoms. Gains in self-efficacy and ER, improvement in grief, and improvement in psychopathology symptoms did not correlate significantly with the event, time since the event, age, or gender. Therefore, because of the rather small sample size, we did not include covariates in the mediation models. Regression analyses showed that the intervention predicted a significant increase in ER (β=.33; *P*=.001) and self-efficacy (β=.30; *P*=.002; Table 3).

**Table 2:** Correlations between improvements in grief and psychopathology, gains in coping self-efficacy, gains in emotion regulation, and baseline variables.

Parameters	TRIG^a^ change	BSI^b^ change	TRIG before	BSI before	CSE^c^ change	ER^d^ change	Event^e^	Time since event	Sex
TRIG change	—^f^	—	—	—	—	—	—	—	—
BSI change	0.35^g^	—	—	—	—	—	—	—	—
TRIG before	0.29^h^	0.17	—	—	—	—	—	—	—
BSI before	0.04	0.50^g^	0.48^g^	—	—	—	—	—	—
CSE change	0.28^h^	0.36^g^	0.01	0.05	—	—	—	—	—
ER change	0.48^g^	0.38^g^	0.24^i^	0.14	0.45^g^	—	—	—	—
Event^e^	0.02	−0.11	0.15	−0.08	−0.15	−0.11	—	—	—
Time since event	0.04	0.02	−0.12^j^	−0.04	0.14	0.18	−0.13^i^	—	—
Sex	−0.15	−0.14	−0.07	−0.14	0.12	0.02	−0.12	−0.08	—
Age	0.06	0.01	0.05	−0.02	−0.06	0.01	0.48^g^	0.16^i^	0.10

^a^TRIG: Texas Revised Inventory of Grief.

^b^BSI: Brief Symptom Inventory.

^c^CSE: coping self-efficacy.

^d^ER: emotion regulation.

^e^Event: 0=separation or divorce, 1=spousal bereavement.

^f^Not applicable.

^g^*P*<.001.

^h^*P*<.01.

^i^*P*<.05.

^j^*P*<.10.

**Table 3:** Results of the mediation analyses.

Analyses						*B*			SE			*P* value			95% CI		β		*R^2^*
**Gains in emotion regulation**																			
	**Direct**																		
		Group			1.83			0.55			.001			0.76 to 2.91		.33		0.107	
**Gains in coping self-efficacy**																			
	**Direct**																		
		Group			1.99			0.66			.002			0.71 to 3.28		.30		0.089	
**Models with one mediator**																			
	**Improvement in grief (TRIG^a^) and emotion regulation**																		
		**Direct**																	
			Group	0.37			0.13			.004			0.12 to 0.63			.26		0.305	
			Gains ER^b^	0.10			0.02			<.001			0.06 to 0.15			.41		0.305	
		**Indirect**																	
			Gains ER	0.19			0.07			.009			0.05 to 0.33			.13		0.305	
	**Improvement in grief (TRIG) and coping self-efficacy**																		
		**Direct**																	
			Group	0.46			0.14			<.001			0.19 to 0.73			.32		0.209	
			Gains CSE^c^	0.05			0.02			.02			0.01 to 0.09			.24		0.209	
		**Indirect**																	
			Gains CSE	0.10			0.05			.06			−0.005 to 0.21			.07		0.209	
	**Improvement in psychopathology (BSI^d^) and emotion regulation**																		
		**Direct**																	
			Group	0.12			0.08			.13			−0.04 to 0.28			.15		0.183	
			Gains ER	0.05			0.02			<.001			0.02 to 0.08			.35		0.183	
		**Indirect**																	
			Gains ER	0.09			0.04			.02			0.02 to 0.17			.12		0.183	
	**Improvement in psychopathology (BSI) and coping self-efficacy**																		
		**Direct**																	
			Group	0.13			0.08			.10			−0.02 to 0.29			.16		0.181	
			Gains CSE	0.04			0.01			<.001			0.02 to 0.07			.35		0.181	
		**Indirect**																	
			Gains CSE	0.08			0.04			.02			0.02 to 0.16			.10		0.181	
**Models with 2 parallel mediators**																			
	**Improvement in grief (TRIG)**																		
		**Direct**																	
			Group	0.37			0.13			.005			0.11 to 0.63			.26		0.297	
			Gains ER	0.10			0.03			<.001			0.04 to 0.15			.39		0.297	
			Gains CSE	0.01			0.02			.73			−0.04 to 0.05			.04		0.297	
		**Indirect**																	
			Gains ER	0.19			0.07			.008			0.03 to 0.33			.13		0.297	
			Gains CSE	0.02			0.05			.73			−0.08 to 0.11			.01		0.297	
			CSE and ER	0.18			0.08			.02			0.05 to 0.34			.14		0.297	
	**Improvement in psychopathology (BSI)**																		
		**Direct**																	
			Group	0.10			0.08			.21			−0.06 to 0.26			.13		0.177	
			Gains ER	0.03			0.02			.05			0.001 to 0.07			.24		0.177	
			Gains CSE	0.03			0.01			.04			−0.001 to 0.06			.23		0.177	
		**Indirect**																	
			Gains ER	0.06			0.04			.09			−0.01 to 0.14			.08		0.177	
			Gains CSE	0.06			0.03			.11			−0.01 to 0.14			.07		0.177	
			CSE and ER	0.12			0.04			.003			0.04 to 0.19			.15		0.177	

^a^TRIG: Texas Revised Inventory of Grief.

^b^ER: emotion regulation.

^c^CSE: coping self-efficacy.

^d^BSI: Brief Symptom Inventory.

### Mediation Analyses

#### Overview

**Figure 1:**
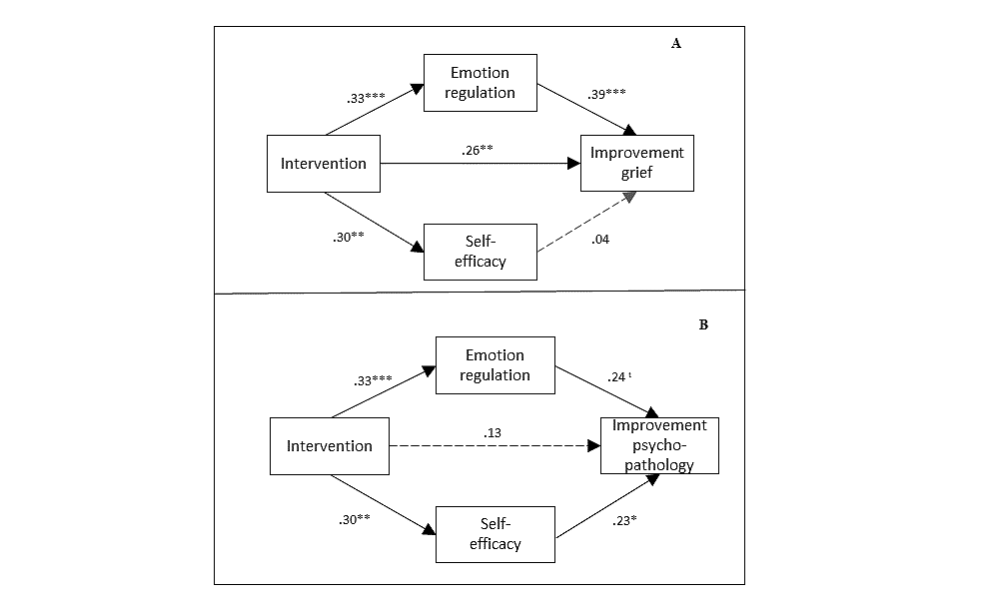
Path models for improvement in grief (A) and psychopathology (B); direct effects, standardized coefficients. *P<.05; **P<.01; ***P<.001; t: *P*=.054.

To investigate whether gains in ER and loss-related CSE were mechanisms of change, we used mediation models for improvements in grief and psychopathology symptoms. Table 3 presents the results of the mediation analyses, including the indirect effects. Figure 1 depicts the path models with direct paths for the models with the simultaneous inclusion of both mediators.

#### Mediation Models With a Single Mediator

*Improvement in grief* was associated with gains in ER (β=.41; *P*≤.001) and gains in CSE (β=.24; *P*=.02). Only ER showed a significant indirect path with a medium effect size (β=.13; *P*=.009). The indirect effect for CSE showed a small to medium–sized effect but did not reach the significance level (β=.07; *P*=.06).

*Improvement in psychopathology symptoms* was related to gains in ER and loss-related self-efficacy (ER: β=.35, *P*≤.001; CSE: β=.35, *P*≤.001). ER and loss-related self-efficacy functioned as mediators with medium effect sizes (indirect effects: ER: β=.12, *P*=.02; CSE: β=.10, *P*=.02).

#### Models With Simultaneous Parallel Mediators

Including ER and loss-related CSE simultaneously in the analyses, the relative strength of both mediators was investigated in exploratory models. *Improvement in grief* was associated with gains in ER (β=.39; *P*≤.001) but not with gains in self-efficacy (β=.04; *P*=.73; Figure 1). Only ER mediated the association between taking part in the intervention and improvement in grief (indirect effect: β=.13; *P*=.008). The path from intervention to improvement in grief remained significant (β=.26; *P*=.005). The total indirect effect was significant and showed a medium effect size (*β*=0.14; *P*=.02).

*Improvement in psychopathology symptoms* was significantly predicted by gains in loss-related CSE (β=.23; *P*=.04; Figure 1). ER showed a similar effect size but did not reach the significance level (β=.24; *P*=.05). The total indirect effect was significant (β=.15; *P*=.003) but not for the specific indirect paths. However, post hoc Monte Carlo power analysis for indirect effects indicated that the models with 2 parallel mediators did not have enough power to detect specific indirect effects apart from the indirect path from ER on the improvement of grief (Multimedia Appendix 1).

## Discussion

### Principal Findings

This study examined ER and loss-related CSE as putative mediators for improvement in grief and psychopathology symptoms in an internet intervention for older adults after spousal bereavement, separation, or divorce. The results suggested that the cognitive behavioral intervention called LIVIA increased both ER and CSE, which correlated with improvements in grief and psychopathology symptoms. Mediation models confirmed ER and loss-related CSE as mediators of improvements in psychopathology. For improvements in grief, only ER showed a significant indirect effect, whereas CSE showed a trend. A model that simultaneously included both predictors suggested that only gains in ER mediated the association between participating in LIVIA and improvement in grief. Only the total indirect effect was significant in the model for improvement in psychopathological symptoms.

Our findings are in line with previous studies showing that ER was linked to treatment outcomes such as depression, negative affect, and other psychological disorders [23-25]. Our results are also consistent with findings that CSE was associated with lower emotional distress, higher psychological well-being in widows, and lower grief symptoms in students who had lost a close person in a university campus shooting [18,19].

Extending existing knowledge, our study investigated ER and loss-related CSE as putative mediators of improvements in grief and psychopathological symptoms after the loss of a spouse. The mediation models with single mediators confirmed a specific direct and indirect effect of gains in ER on improvement in grief and psychopathology symptoms. This suggests that ER was a mediator and potential mechanism of change. Gains in CSE showed significant direct effects on grief and psychopathology symptoms and a significant indirect effect on psychopathology symptoms. However, there was only a marginal indirect effect on grief symptoms. A power analysis revealed a power of 0.59 for finding a significant indirect path via CSE. This effect may have been significant in a larger sample.

Exploratory mediation models with the simultaneous inclusion of ER and loss-related CSE aimed to investigate the specificity and relative strength of these variables as parallel mediators. The results confirmed the importance of ER for improvement in grief and suggested that loss-related CSE was less relevant. However, the models were underpowered to detect any significant indirect effects. This also impedes a clear interpretation of the model for improvement in psychopathology symptoms.

Referring to the dual process model of coping with bereavement [28], in addition to loss-related CSE, ER skills may be especially important for loss-oriented work, such as experiencing the pain of loss. An improvement in ER skills such as self-soothing or cheering oneself up may make these processes more tolerable as individuals can regulate overwhelming or more persistent emotions. In addition, oscillating between loss- and restoration-oriented tasks may be fostered by better ER skills. Our findings underline the crucial importance of ER for grief interventions and corroborate previous studies suggesting that ER was a mediator in an internet intervention for stress management [27]. Nevertheless, prolonged grief assessed with the TRIG has a strong separation distress component, which can be regarded as an ER problem. Therefore, these results do not necessarily generalize to persistent complex bereavement disorders, which also include avoidance symptoms and impairment in social, occupational, and other areas of life.

ER and loss-related CSE resulted in significant specific indirect effects on improvements in psychopathology symptoms, suggesting that both may be mediators for improvement in psychopathology. This is in line with Benight et al [20], who found that a change in CSE predicted a decrease in posttraumatic stress. High loss-related CSE may facilitate mainly restoration-oriented tasks such as addressing all the changes caused by the loss and creating a new life without the spouse, which may be perceived as very demanding. The belief in the ability to cope with these tasks may render them less threatening and alleviate stress and anxiety, as well as promote engagement in coping behavior and sustain coping efforts [17]. In addition, CSE may foster a sense of autonomy, self-determination, purpose in life, and perceived environmental mastery, which leads to less avoidant behavior and less aversive rumination [18]. Thus, loss-related CSE could promote a positive adaptation to life without a partner and decrease grief.

A further difference between the mediation models for grief and psychopathology was the significant direct effect of taking part in LIVIA and improvement in the outcome variables. Interestingly, and in contrast to the model for grief, no significant direct path existed from the intervention to improvements in psychopathological symptoms. In addition, improvement in grief was greater than that in psychopathology symptoms (*d*=0.81 vs *d*=0.39) [33], and the amount of explained variance was larger for grief than for psychopathology symptoms (30% vs 13%). This may reflect the fact that grief was a major focus of LIVIA, which was specifically developed for older adults who had lost their spouse through bereavement or divorce. For example, a module focused on positive social relationships, which may be more important for improvement in grief than for psychopathology symptoms. Thus, improvement in social support might be an additional mediator for the improvement in grief.

Exploratory mediation models with the simultaneous inclusion of ER and CSE suggested that ER was more important for improvement in grief than CSE, whereas both mediators showed similar effects for improvement in psychopathology. Regarding the interplay between ER and self-efficacy in grief processing, one can speculate that ER may be a more fundamental process than loss-related CSE as a social cognitive variable. The ability to modify negative emotions seems to have a positive effect on loss-related CSE. As Bandura [16] pointed out, emotional arousal can reduce self-efficacy in threatening situations as high arousal can debilitate performance. For example, among combat veterans, ER difficulties had only an indirect effect on a lower quality of life and higher posttraumatic stress symptom severity via lower CSE [43].

### Limitations

Our study has several limitations. A meta-analysis demonstrated that web-based interventions increased disease-specific but not general self-efficacy, and context-specific measures are assumed to be more predictive of adjustment to stress [16]. As there are no psychometrically validated scales for assessing ER and loss-related CSE after bereavement and separation or divorce, we created 3 contextualized items to measure ER and loss-related CSE, specifically in the context of spousal bereavement and separation or divorce and the content of LIVIA. Furthermore, we aimed to keep the questionnaires short in order to not overburden the participants, which could have increased the attrition rate.

As a further limitation, we only had 2 measurement points during the 10-module intervention (ie, preintervention and postintervention) and then computed the difference scores for gains in ER and CSE. Thus, we assessed only changes in ER and CSE over the whole intervention, and thus, there was some temporal overlap in the measurement of the mediators and outcome variables. Therefore, results of the mediation analyses should be interpreted with caution. A fine-grained temporal design may also be able to more accurately detect the temporal sequence of change and the interplay of mediators and thus disentangle the mechanisms of change. However, the speed and shape of change are not necessarily linear, and sudden gains or losses may occur (for more details, see the study by Aderka et al [44]). Thus, the appropriate time point for assessing the mediators for capturing these changes may be difficult to determine, and the temporal associations between changes in the mechanism and changes in outcomes may be hard to disentangle [45]. Moreover, the sample size of 100 participants limited the number of variables in the models, precluded a more detailed analysis of the interplay between potential moderators and mediators, and led to power issues for the models with 2 parallel mediators. Moreover, the sample included only 20% (20/100) of widowed individuals, which precluded separate models for widowed participants.

Considering these limitations, the results of this study must be replicated and extended by using larger samples and more measurement points. Further research should use validated measures for ER and loss-related CSE and investigate whether the greater relative importance of ER compared with CSE is specific to prolonged grief symptoms or whether it also generalizes to distress-related disorders and other psychological disorders such as anxiety disorders. In addition, other potential mediators such as social support could be examined together with ER and CSE.

Despite these limitations, the findings of this study have several clinical implications. Gains in ER and loss-related CSE are promising targets for improving internet interventions and probably also face-to-face interventions for coping with the consequences of spousal bereavement, separation or divorce. Gains in ER and self-efficacy could be conceptualized as common factors in psychotherapy related to improved behavioral regulation and changing expectations of personal effectiveness [15]. Thus, one could hypothesize that ER and CSE are also mechanisms of change in interventions for different psychological disorders and in the promotion of psychological well-being in general.

Depending on the problems of the participants, more specific modules for ER or CSE could be added to the intervention. Techniques aimed at strengthening ER include, for example, emotional skills training. CSE could be bolstered by training in adaptive coping strategies, mastery experiences, or reappraising emotional and physiological reactivity [16]. These techniques could be integrated as additional modules or replace less effective modules. Alternatively, users of existing interventions could be advised to spend more time and effort on their respective modules.

### Conclusions

This study is one of the few to investigate mediators in internet interventions and the first to examine emotional and cognitive processes as mediators in grief processing after spousal bereavement, separation, or divorce. Our findings suggest that ER and loss-related CSE mediated treatment outcomes and are promising therapeutic targets for improving grief and psychopathology symptoms in internet interventions.

ER and CSE should be examined as transdiagnostic or disorder-specific putative mediators in internet interventions for other disorders.

## Appendix

Multimedia Appendix 1 Exploratory and confirmatory factor analyses for emotion regulation and loss-related coping self-efficacy and Monte Carlo power analysis for indirect effects.

Multimedia Appendix 2 CONSORT-eHEALTH checklist (V 1.6.1).

## Abbreviations

CBT: cognitive behavioral therapy
CSE: coping self-efficacy
ER: emotion regulation
TRIG: Texas Revised Inventory of Grief
