# Supplementary material for: The Role of Emotion Regulation and Loss-Related Coping Self-efficacy in an Internet Intervention for Grief: Mediation Analysis
Source: JMIR Ment Health. 2022 May 6;9(5):e27707. doi: 10.2196/27707 (PMC9123547; doi:10.2196/27707)
Supplement: Multimedia Appendix 1 [file mental_v9i5e27707_app1.docx]

**Multimedia Appendix 1.**  **Exploratory and confirmatory factor analyses for emotion regulation and loss-related coping self-efficacy and Monte Carlo power analysis for indirect effects**

**1. Exploratory and confirmatory factor analyses for emotion regulation and loss-related coping self-efficacy**

Loss-related coping self-efficacy was defined as expectancies to be able to cope with the death of the partner. Emotion regulation assessed the ability to foster positive emotions and thoughts. Both were targets of LIVIA and potential mechanisms of change. For each module of LIVIA, we formulated session-related outcomes that were supposed to capture the therapeutic target of the module and/or potential mechanisms of change. They were assessed at the beginning and the end of the intervention.

We performed an exploratory factor analysis with a Promax rotation including all session-related items that may capture mechanisms of change, i.e., emotion regulation and loss-related coping-self-efficacy apart from the item “I can cope with the pain of loss”. As this item may be associated with emotion regulation as well as loss-related coping-self-efficacy, we excluded this item from the analyses based on these theoretical thoughts.

The comparison of the models based on the Chi-square and the degrees of freedom indicated that a two-factor model was preferred over a one-factor or three-factor model. Factor loadings of the two-factor solution are presented in Table 1.

**Table 1.**

Results of the exploratory factor analysis using a promax rotation

| *Item* |  | *f1* | *f2* |  |
| --- | --- | --- | --- | --- |
| 3 | I am confident in my ability to cope with the loss of my partner. | 0.84 | 0.08 |  |
| 4 | I am prepared to do the things necessary to cope with my loss. | 0.92 | -0.08 |  |
| 5 | I have great influence in coping with my loss. | 0.54 | 0.15 |  |
| 7 | I can take care of my own well-being. | 0.34 | 0.58 | CL |
| 8 | I can cheer myself up. | -0.01 | 0.92 |  |
| 9 | I can influence my thoughts and feelings in a positive way. | 0.06 | 0.87 |  |
| 12 | I can manage my life without a partner in a way that makes me feel good. | 0.47 | 0.37 | CL |

Note: CL = cross-loading

Item 12 was not included in the confirmatory factor analysis as it loaded substantially on both factors. Item 7 also showed a cross-loading, however, the loading on f2 was dominant. Therefore, we included it in the emotion regulation factor, while the loss-related self-efficacy factor remained unchanged.

Confirmatory factor analyses indicated that all items loaded significantly and substantially on the respective factors and indicated that a two-factor solution was preferable over a one-factor solution (1 factor model: CFI = .84, TLI = .74, RMSEA = .273; 2 factor model: CFI = .95, TLI = .91, RMSEA = .159). The results of the confirmatory factor analysis with two factors are reported in Table 2.

**Table 2**

*Results of the confirmatory factor analysis with two factors*

| *Item* | *Standardised Estimate* | *S.E.* | *P* |
| --- | --- | --- | --- |
| *Factor Loss-related coping self-efficacy* | | | |
| 3. I am confident in my ability to cope with the loss of my partner. | .93 | .03 | < .001 |
| 4. I am prepared to do the things necessary to cope with my loss. | .83 | .04 | < .001 |
| 5. I have great influence in coping with my loss. | .67 | .03 | < .001 |
| *Factor Emotion regulation* | | | |
| 7. I can take care of my own well-being. | .84 | .04 | < .001 |
| 8. I can cheer myself up. | .90 | .03 | < .001 |
| 9. I can influence my thoughts and feelings in a positive way. | .91 | .03 | < .001 |

**2. Monte Carlo power analysis for indirect effects**

We performed post-hoc Monte Carlo power analysis for indirect effects for the given mediation models with one and two parallel mediators using http://marlab.org/power_mediation/. [1] Results showed that the model with loss-related coping self-efficacy as a mediator for the improvement of grief was underpowered, see Table 2. For the models with two parallel mediators, all paths were underpowered apart from the estimation of the indirect effect via emotion regulation on improvement of grief.

**Table 3**

*Results of Monte Carlo power analysis for indirect effects for the mediation models*

| *Mediator* | *Grief* | *Psychopathology* |
| --- | --- | --- |
| *Models with one mediator* | | |
| Emotion regulation (ER) | .93 | .86 |
| Loss-related coping self-efficacy (CSE) | .59 | .80 |
| *Models with two parallel mediators* | | |
| Indirect effect via ER | .86 | .51 |
| Indirect effect via CSE | .05 | .42 |

**Reference**

Schoemann, A. M., Boulton, A. J., & Short, S. D. (2017). Determining power and sample size for simple and complex mediation models. *Social Psychological and Personality Science*, *8*(4), 379-386.
